# Does It Really Matter? Separating the Effects of Musical Training on Syntax Acquisition

**DOI:** 10.3389/fpsyg.2012.00543

**Published:** 2012-12-13

**Authors:** Garvin Brod, Bertram Opitz

**Affiliations:** ^1^Center for Lifespan Psychology, Max Planck Institute for Human DevelopmentBerlin, Germany; ^2^Department of Psychology, Saarland UniversitySaarbrücken, Germany; ^3^School of Psychology, University of SurreyGuildford, UK

**Keywords:** syntax acquisition, hierarchical syntax, L2 learning, musical training, transfer effects

## Abstract

The possible transfer of musical expertise to the acquisition of syntactical structures in first and second language has emerged recently as an intriguing topic in the research of cognitive processes. However, it is unlikely that the benefits of musical training extend equally to the acquisition of all syntactical structures. As cognitive transfer presumably requires overlapping processing components and brain regions involved in these processing components, one can surmise that transfer between musical ability and syntax acquisition would be limited to structural elements that are shared between the two. We propose that musical expertise transfers only to the processing of recursive long-distance dependencies inherent in hierarchical syntactic structures. In this study, we taught fifty-six participants with widely varying degrees of musical expertise the artificial language BROCANTO, which allows the direct comparison of long-distance and local dependencies. We found that the quantity of musical training (measured in accumulated hours of practice and instruction) explained unique variance in performance in the long-distance dependency condition only. These data suggest that musical training facilitates the acquisition specifically of hierarchical syntactic structures.

## Introduction

Several recent studies have examined transfer effects of musical training to language proficiency. In a well received article, Slevc and Miyake (2006) examined the relationship between proficiency in a second language (L2) and musical ability in Japanese adult learners of English living in the USA. By using a regression analysis, which included factors such as age of arrival, length of residence, and short-term memory capacity, they demonstrated that musical ability explains unique variance for both receptive and productive phonological ability in the L2. However, musical ability did not account for unique variance in L2 syntax proficiency (although the correlation between musical ability and L2 syntactic proficiency was significant). Apart from this study, positive transfer effects of musical training on language processing have for instance been shown for prosody (Besson et al., 2007), linguistic pitch patterns (Wong et al., 2007), verbal working memory (Ho et al., 2003), and improved reading skills (Moreno et al., 2009). As has been argued previously, cognitive transfer requires overlap of processing components and of brain regions involved in these processing components (Jonides, 2004). Regarding the organization of linguistic syntax upon which cognitive processes may act, Chomsky and Schutzenberger (1963) proposed a distinction between a lower-level grammar, which they called Finite state grammar (FSG), and higher level grammar, named Phrase Structure Grammar (PSG). The FSG follows linear organizational principles using only transitional dependencies between adjacent elements to generate the sequence. In contrast, the PSG contains additional hierarchical organizational principles using center-embedded structures and recursion, thereby generating conditional non-adjacent or long-distance dependencies. To illustrate the difference between the two, consider the dependency between the subject “Anna” and the verb “needs” in the simple sentence “Anna needs the salt” as compared to “Anna, the girl living next door, needs the salt.” Likewise, to analyze harmonic progressions in tonal music on different structural levels, Rohrmeier (2011) proposed a hierarchically organized set of rules in analogy to linguistic syntax using parse trees. He pointed out that Western tonal music and linguistic syntax share some key organizing principles, e.g., recursivity, hierarchical organization, and long-distance dependencies, whereas other principles, like valence or case assignment, seem to be exclusively found in language (for an extensive argument, see Rohrmeier, 2011). In line with this account, a recent behavioral study revealed that reading times for syntactically, but not for semantically, irregular words were increased when presented together with a music-syntactic violation (Slevc et al., 2009). Thus it could be argued that the computational implementation of analogous hierarchical organization in tonal harmony vs. linguistic syntax might be reflected in shared cognitive processes (although a one-to-one mapping cannot be assumed; Rohrmeier, 2011). A number of studies employing various methods support the prediction that music and language share neural resources for syntax integration (Shared syntactic integration resource hypothesis, Patel, 2003). Using MEG (Maess et al., 2001) and fMRI (Tillmann et al., 2006) it was demonstrated that processing musical syntax is linked to the inferior frontal cortex, especially Broca’s Area (and its right hemisphere homolog, see Fadiga et al., 2009, for a recent review). Opitz and Friederici (2007) were among the first to specifically investigate the neural underpinnings of the hierarchical organization of linguistic syntax by means of fMRI. Using an artificial language that allowed the direct comparison between local phrase structure dependencies and long-distance dependencies (i.e., hierarchical rules), they found that activation in the left ventral premotor cortex (vPMC) and in the hippocampus was related to the local character of rule change. Sentences containing violations of long-distance dependencies activated the opercular part of the inferior frontal gyrus (Broca’s Area). These results are in accordance with earlier studies reporting activation in Broca’s Area during the learning of long-distance dependencies (Tettamanti et al., 2002; Musso et al., 2003; Opitz and Friederici, 2003) and activation in the vPMC for the learning of local structures (Friederici et al., 2003; see also Opitz and Kotz, 2012). Taken together, the results of all the aforementioned studies may indicate that the processing of adjacent and long-distance dependencies relies on different brain areas, with common involvement of Broca’s Area in the processing of both tonal and linguistic hierarchical structures. Given this evidence that cognitive mechanisms and brain regions involved in processing tonal and linguistic structures at least partially overlap, the aim of the present study was to investigate whether there is a positive transfer effect for syntax acquisition in a second language due to musical expertise. For this purpose, a modified version of a highly controlled artificial language (BROCANTO) was employed that allows direct comparisons between local and long-distance dependencies, whilst keeping all other aspects of language processing constant. These types of dependencies have clear parallels within natural language structure. Thus, the relationship between words within phrases and between different phrases themselves can be described using local dependencies, whereas embeddings or recursions rely on long-distance dependencies. Moreover, the modified version of BROCANTO used in the present experiment meets the universal requirements of natural language and can be viewed as a small but expandable section of a fully fledged language comparable to natural languages (Friederici et al., 2002). This view was corroborated by a recent study using an artificial language learning task involving local and long-distance dependency structures similar to BROCANTO (Misyak and Christiansen, 2012). This study demonstrated that increased accuracy in detecting local dependencies correlated only with the comprehension scores for sentences involving local dependency structures such as homonyms with noun/verb resolution [e.g., “the student needs (were not being met)/(to be more focused),” Misyak and Christiansen, 2012]. The comprehension of Subject-Object Relative Clauses, however, correlated with the participants’ performance for both local and long-distance dependencies. These results, although correlational in nature, make it plausible that sufficiently language-like artificial grammars (like BROCANTO) are predictive of at least some aspects of L2 syntax learning and therefore to some extent rely on at least similar learning mechanisms (see Conway and Pisoni, 2008, for a detailed discussion). Based on the available evidence outlined above, we hypothesize that there is a partial (rather than overall) positive transfer effect for syntax acquisition in musicians. We argue that people with a high amount of musical training should have an advantage in the acquisition of long-distance dependencies, but would not differ from people with a low amount of musical training with respect to the acquisition of local dependency structures.

## Materials and Methods

### Participants

To test a sufficient amount of musically trained subjects, participants were recruited both at Saarland University and the University of Music, Saarland. All fifty-six participants were native German speakers and gave their written, informed consent prior to testing. Musical training was assessed via a detailed questionnaire (see Appendix) evaluating training intensity (hours of musical training) by age period. Participants were asked to estimate the amount of their musical training within age periods of 3 years (e.g., age 4–7. 7–10,…), along with mean training hours per day and per week. They were asked to list instruments played and kind of musical experience (e.g., choir, orchestra, music education for young children) during the different time periods. This procedure was meant to provide them with cues to improve their ratings. The age at inception of musical training, whether the participants possessed absolute pitch, and the number of spoken languages/bilingual environment were assessed as well. Visual inspection revealed no link between the number of spoken languages and performance in the AGL task or musical training hours. One participant possessed perfect pitch; three participants were raised in bilingual environments. Data from two students had to be excluded from all analyses either due to a computer malfunction or due to a misunderstanding of the instructions. Another nine subjects were excluded because they consistently did not perform above chance level (computed across both conditions). As previously suggested (Opitz and Friederici, 2007), a relatively high proficiency seems to be a prerequisite for transfer effects to occur. Ultimately, the data from 45 students (mean age 23.1, range 19–31, 30 females) entered the main analysis. A cognitive test battery was administered in a second session at a later date to 36 participants (nine participants could not be tested due to unavailability). As phonological short-term memory capacity has shown systematic relations to L2 syntax acquisition (Ellis and Sinclair, 1996), participants underwent a digit-span task adapted from the German adaptation of the WAIS-III (von Aster et al., 2006; mean performance: 9.56, range: 5–13, SD: 1, 96). In addition to the maintenance component of working memory, the explicit manipulation of verbal information in working memory was addressed by means of the Adaptive Digit Ordering Test (A-DOT, Werheid et al., 2002; mean performance: 9.64, range: 5–13, SD: 2.11). For the A-DOT test participants had to verbally repeat numbers spoken by the experimenter, but in ascending order (e.g., 3-1-2 → 1-2-3). A trail-making test of perceptual speed, the “Zahlen-Verbindungs-Test,” (mean performance: 53.76 s, range: 37.5–84 s, SD: 9.18; Oswald and Roth, 1987) was incorporated. The importance of assessing perceptual speed was demonstrated by Helmbold et al. (2005): they compared music students with students of other subjects (mostly of psychology) from German universities and found no differences in the structure of intelligence or in specific aspects of mental abilities, except for flexibility of closure and perceptual speed. Furthermore, this trail-making test was shown to be a good screening measure of general fluid intelligence (Vernon, 1993).

### Stimuli

**Figure 1:**
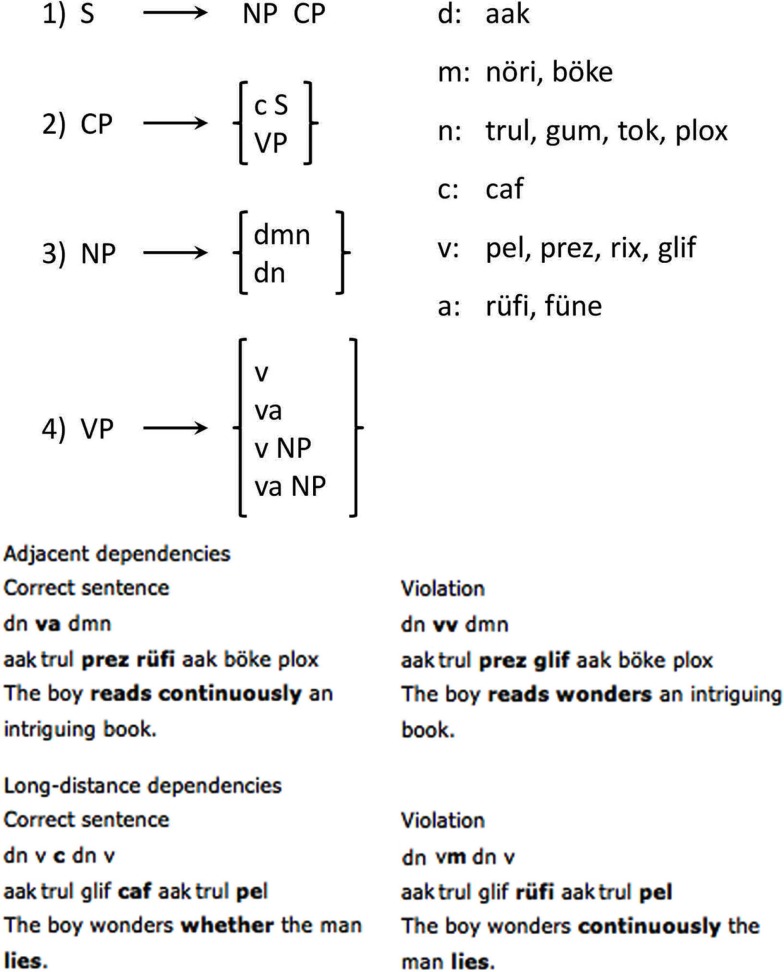
**Schematic illustration of the rules underlying the artificial language BROCANTO**. Capital letters represent non-terminals: NP, noun phrase; CP, complementizer phrase; VP, verb phrase. Terminal symbols are depicted as lower case letters: d, determiner; m, modifier (adjective); n, noun; v, verb, a, adverb; c, complementizer. The vocabulary is depicted on the right. Below, examples of adjacent and long-distance dependencies in BROCANTO along with literal English counterparts are given. The respective dependencies are marked in bold.

The stimuli were quite similar to those described in Opitz and Friederici (2007), i.e., they followed the same rule system and were comprised of the same vocabulary, but contained additional sentences. Thus, 200 sentences were formulated, half composed of only local dependencies and the other half also including long-distance dependencies. These long-distance dependencies were derived according to a recursive rule (see Figure 1). Additionally 200 sentences, again divided into two equal parts of local/long-distance dependency structures, were formulated containing a severe syntactic violation. The non-grammatical version of the long-distance dependency condition was rendered ungrammatical by a second verb phrase at the end of the sentence, which would only be licensed after a complementizer element. In case of local dependencies, ungrammaticality is realized by two successive elements of the same class (terminals as well as non-terminals), which is not allowed by the grammar (see Opitz and Friederici, 2007 – for further examples).

### Procedure

The experiment was divided into alternating learning and test blocks. During the learning blocks, participants were given observation training on 20 correct sentences presented for 7 s each on a computer screen (half of them containing long-distance dependencies) and were instructed to extract the underlying grammatical rules. During the test blocks, 20 new sentences that were either grammatical (half of the sentences) or ungrammatical appeared one after another for 7 s on the screen. Participants were asked to judge the correctness of these sentences according to the grammar they learned, implemented by a six point rating-scale allowing the participants to additionally state confidence in their judgments (ranging from 1, “surely grammatically correct” to 3, “rather grammatically correct” for the supposedly correct sentences and from 4, “rather grammatically incorrect” to 6, “surely grammatically incorrect” for the supposedly incorrect sentences). A rating-scale was used instead of a binary decision to evaluate the participants’ response profile (e.g., only low confidence ratings). The test blocks were equally divided into correct and incorrect sentences of both local and long-distance dependency structures. Participants were instructed to respond while the sentence was on the screen, i.e., within 7 s. In total, 15 learning blocks and 15 test blocks were presented.

### Data analysis

The response profile for both syntactic structures was first evaluated using a chi-square test on the number of responses for each confidence level. This analysis revealed no differences between the two types of linguistic structure [*χ*^2^(5) = 6.9, *p* > 0.23]. For further analysis, responses were collapsed across several confidence levels to represent grammatical responses (i.e., a 1, 2, or 3 confidence rating) or non-grammatical responses (i.e., a 4, 5, or 6 confidence rating), respectively. This dichotomization corresponds to the instructed meaning of the numbers (see Procedure). Pr [p(hit)-p(false alarm)] was calculated for the beginning (collapsed across test blocks 1 and 2), the middle (test blocks 7 and 8), and the final phase (test blocks 14 and 15) of the learning session. To test whether the participants gained more expertise in BROCANTO during the experiment, a repeated-measures analysis of variance (ANOVA) was computed that included within-subject factors of syntax type (local vs. long-distance dependencies) and time (beginning, middle, and final phase of the experiment). To confirm this approach, a separate analysis with the total 15 blocks was performed. As the amount of deliberate practice and not the mere duration has previously been shown to best account for musical ability (Ericsson et al., 1993), musical expertise was quantified by the accumulated training hours until the age of 19 (for detailed arguments in favor of this estimate see Discussion). Musical expertise was then correlated across participants with the achieved level of proficiency in BROCANTO. Based on the results of this correlation, a hierarchical regression analysis was computed which in addition to musical expertise contained the above described measures of working memory and perceptual speed as regressors.

### Results

**Figure 2:**
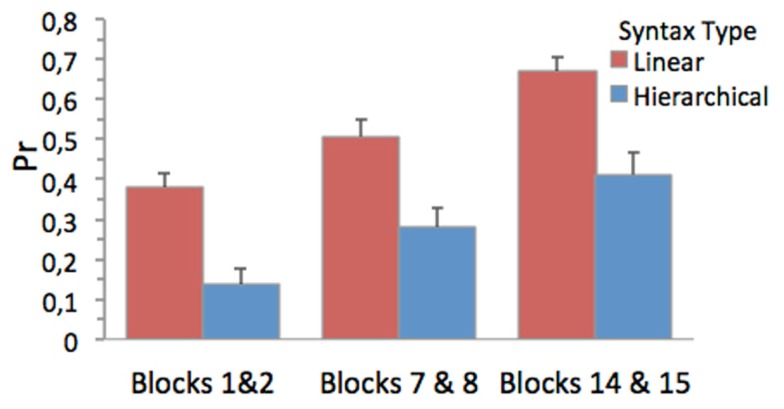
**Discriminability between correct and in correct sentences measured as Pr [p(hit)-p(false alarm)] and averaged for the initial (Blocks 1 and 2), the middle (Blocks 1 and 2), and the final two test blocks (Blocks 14 and 15) and the within-subject factor syntax type**. Bars shows means errors bars indicate mean 1.0 standard errors (SE).

As apparent from Figure 2, participants increased their knowledge of both local and long-distance dependency structures of BROCANTO in the course of the experiment. This was confirmed by the repeated-measures ANOVA showing a main effect of time [*F*(2, 43) = 29.86, MSE = 0.06, *p* < 0.001, $\eta_{p}^{2}$ = 0.40] qualified by a linear trend [*F*(1, 44) = 55.17, MSE = 0.07, *p* < 0.001, $\eta_{p}^{2}$ = 0.56] in the absence of an interaction between time and syntax type [*F*(2, 43) = 0.18. MSE = 0.04, n.s]^1^. As indicated by a main effect of syntax type [*F*(1, 44) = 47.92, MSE = 0.08, *p* < 0.001, $\eta_{p}^{2}$ = 0.52] local dependency structures were easier to acquire than long-distance dependency structures.

**Figure 3:**
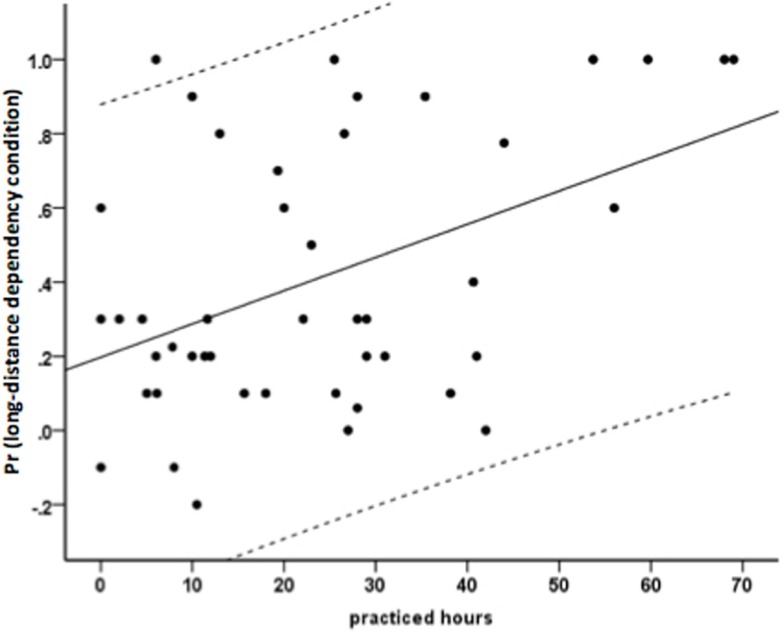
**Relationship between cumulated hours of musical training until 19 and the achieved level of syntax proficiency (measured as Pr) for the long-distance dependency condition**. There is a significant positive correlation between the two variables (*r*=0.46, *p* <0.01, indicated by the solid line). The dashed lines indicated the 95% confidence interval.

Furthermore, a correlation analysis (see Table 1 for complete results) revealed a significant relationship between hours of musical training until 19 and achieved level of syntax proficiency for the long-distance dependency condition (*r* = 0.45, *p* < 0.01, see Figure 3), but not for the local one (*r* = 0.03, *p* > 0.8). To further validate this finding an additional Spearman’s rank order correlation was carried out as this is more robust with respect to outliers. This analysis also revealed a positive relationship between musical training hours and performance in the long-distance dependency condition (*r* = 0.31, *p* < 0.05). To explore whether this relationship persists in the long-distance dependency condition, when other factors shown to be relevant for the acquisition of L2 syntax are considered, a hierarchical regression analysis was conducted (see Table 2 for a summary of the results). This analysis indicates that among the investigated factors, only the working memory capacity explained unique variance of the achieved proficiency in addition to musical training. Furthermore, the same hierarchical regression analysis was performed without the outlier participants. Musical training continues to explain unique variance (*R*^2^ change = 0.13, final β = 0.373, *p* = 0.02). Due to the lack of correlation between musical training and performance in the local dependency condition, there was no indication for a further regression analysis. Despite that, however, we conducted a hierarchical regression analysis in the same manner as for the long-distance dependency condition, which, as expected, did not reach significance in any step and is therefore not reported here.

**Table 1:** **Correlation between the measures of the cognitive test battery and the performance in the linear and hierarchical syntax condition**.

	1	2	3	4	5	6
1. Trail-making test (ZVT)						
2. Wechsler digit-span-test	−0.52**					
3. Digit-ordering test (DOT-A)	−0.55**	0.61**				
4. Practiced hours	−0.14	0.02	0.04			
5. Pr linear condition	−0.02	0.12	0.26	0.03		
6. Pr hierarchical condition	−0.20	0.001	0.28*	0.46**	0.25*	

*p < 0.05, **p < 0.01.

**Table 2:** **Hierarchical regression analysis**.

Step and independent variable	*R*^2^	*R*^2^ change	Df	*F*	Final β
Step 1: trail-making test (ZVT)	0.04	0.04	1,34	1.34	−0.03
Step 2: digit-span-test	0.05	0.01	1,33	0.89	−0.28
Step 3: digit-ordering-test (DOT-A)	0.14	0.09^†^	1,32	1.68	−0.41*
Step 4: practiced hours	0.32	0.19**	1,31	3.99	−0.44**

Final β = β in the fourth step. ^†^ = *p* < 0.10 * = *p* < 0.05, ** = *p* < 0.01.

*Dependent variable: Pr-values for the hierarchical syntax condition*.

## Discussion

The aim of this study was to explore whether musical ability can influence the acquisition of syntactical structures in a L2. As hypothesized, hours of musical training until the age of 19 was correlated with performance in the long-distance dependency condition, but not with performance in the local dependency condition. Furthermore, when other relevant factors were included, musical training until the age of 19 explained unique variance in the hierarchical syntax condition only.

Hours of musical training until age 19 was chosen as the key measure of musical expertise for several reasons: we chose training hours instead of training years because our goal was to assess the accumulated training time, which cannot be equated with the mere length of the interval during which the training occurred. This reasoning was adopted from findings by Ericsson et al. (1993), who have shown that performance differences are closely related to the amount of deliberate practice, particularly during childhood. This finding holds even among highly proficient performers (violin students were used in their study). Other common measures of musical training like years of training or the age at inception of musical training seemed inappropriate because our participants were chosen to represent widely varying degrees of musical expertise (including “one year during childhood” and some participants who stopped playing an instrument during puberty). Therefore, these other measures would have overestimated the musical expertise in the present sample. Moreover, *post hoc* analyses revealed that our results do not depend on the choice of cut-off, as the correlation between performance in the long-distance dependency condition and accumulated training hours across a wide age range (from 13 to 22 years) did not differ significantly from the correlation with accumulated training hours until 19. Thus, the age of the youngest participant at the day of the experiment (i.e., 19) was chosen as the cut-off. These specific transfer effects could be explained based on the previously mentioned overlap between musical syntax (i.e., tonal harmony) and linguistic syntax, both on the structural level and on the neural resources level. In accordance with this idea, it was recently argued that the behavioral and cerebral features involved in processing of complex auditory material, shared between music and language, are based on common evolutionary roots between both domains (James, 2012).

With respect to the structural level, our findings are in accordance with our hypothesis that musical expertise is a driving factor facilitating the acquisition of long-distance dependency structures, whereas it is not beneficial for the acquisition of local dependencies. The observed positive correlation between hours of musical training and performance for long-distance dependencies only corroborated our view of possible transfer effects of musical expertise on processing hierarchical linguistic structures. Support for our notion comes from a study by Jentschke and Koelsch (2009), who investigated the role of musical training for the development of syntactic processing in children. By comparing the ERP responses to violations of musical or linguistic syntax in 10-to-11-year olds with and without musical training, they revealed that neurophysiological mechanisms underlying syntax processing in music and language are developed earlier, and more strongly, in children with musical training. The sentence stimuli used were passive mode constructions in German. The non-local integration (the Noun is not directly followed by the Verb) can be regarded as more complex in terms of computing syntactic dependencies (Chomsky and Milner, 1963), bearing some similarity to our notion of hierarchy/long-distance dependency. Accordingly, non-local integrations are known to be more difficult to process than local integrations (King and Just, 1991). Moreover, our data complement the findings of Slevc and Miyake (2006), who auditorily presented sentences containing both syntax types. While they, at first sight, revealed a positive correlation (*r* = 0.35, *p* < 0.05) between musical ability and L2 syntax proficiency, this relationship became non-significant after controlling for other relevant variables like age of arrival, length of residence, and language use and exposure. This can be reconciled in accordance with our hypothesis in so far as syntactic hierarchy was not taken into account. Our data suggests that an overall positive transfer effect for syntax acquisition in participants with musical expertise might have been suppressed by the role of local dependencies. Although the artificial language learning approach used here was shown to be predictive of at least some aspects of L2 syntax learning and relies on similar learning mechanisms, whether these findings are directly transferrable to the acquisition of natural languages requires further empirical evidence.

What remains an open question, however, is whether musical training facilitates linguistic and other skills directly or whether this transfer is mediated by domain-general improvements (e.g., in executive functions; Schellenberg and Peretz, 2008). Schön and François (2011) argue for the latter option by saying that musical expertise facilitates sequence learning and extracting regularities. These statistical learning skills are supposed to play a key role in artificial grammar learning (Misyak et al., 2010), which in turn predicts comprehension of natural language sentences (Misyak and Christiansen, 2012). As shown in a recent computational modeling approach (see Rohrmeier and Koelsch, 2012), statistical learning mechanisms, together with massive prior exposure, may also underlie the cognitive processing of musical syntax. Therefore, the observed transfer between musical training and learning of long-distance dependencies in an artificial grammar in our study might be explained by an improvement in probabilistic learning of long-distance dependencies in general.

## Conflict of Interest Statement

The authors declare that the research was conducted in the absence of any commercial or financial relationships that could be construed as a potential conflict of interest.

## Footnotes

^1^The additional analysis, in which the total 15 blocks were entered, revealed comparable results, thus confirming the validity of the data segmentation into three blocks.

## Appendix

### Fragebogen: Musikalisches training *(musical training questionnaire)*

ID:………………………………

Alter (*age*):……………………………….

**Table:**

Haben Sie ein absolutes Gehör?	Ja□	Nein□
*Do you possess perfect pitch?*	Yes□	No□

Im welchem Alter haben Sie angefangen zu musizieren?

At which age did you start to make music?

……………………………………………………………………………………………………

Mit welchem Instrument haben Sie angefangen zu musizieren?

Which was your first musical instrument?

……………………………………………………………………………………………………

Welches ist ihr Hauptinstrument?

What is your main musical instrument?

……………………………………………………………………………………………………

Welche zusätzlichen Instrumente spielen Sie?

Which additional musical instruments do you play?

……………………………………………………………………………………………………

Geben Sie bitte an, wie oft Sie im letzten Jahr trainiert haben:

Please indicate now, how often you did practice during the last year:

Durchschnittliche Anzahl Trainingsstunden pro Tag:

Mean practice hours per day:

……………………………………………………………………………………………………

Durchschnittliche Anzahl Trainingsstunden pro Woche:

Mean practice hours per week:

……………………………………………………………………………………………………

Sind Sie bilingual aufgewachsen?

Have you been raised in a bilingual environment?

……………………………………………………………………………………………………

Welche Fremdsprachen sprechen Sie?

Which foreign languages do you speak?

……………………………………………………………………………………………………

**Table A1:** **Evaluation der Trainingsintensität**. *Evaluation of training intensity*.

Zeitperiode (Jahre)	Durchschnittliche Anzahl Trainingsstunden pro Tag	Durchschnittliche Anzahl Trainingsstunden pro Woche	Gespielte Instrumente
*Time period (years)*	*Mean training hours per day*	*Mean training hours per week*	*Instruments played*
4–7			
7–10			
10–13			
13–16			
16–19			
19–22			
22–25			
25–28			
28–31			
31–34			
34–37			
37–40			
